# Dysregulation of an X-linked primate-specific epididymal microRNA cluster in unexplained asthenozoospermia

**DOI:** 10.18632/oncotarget.18076

**Published:** 2017-05-23

**Authors:** Xingrong Qing, Jian Shi, Tingting Dong, Chunlin Wu, Lian Hu, Honggang Li

**Affiliations:** ^1^ Family Planning Research Institute/Center of Reproductive Medicine, Tongji Medical College, Huazhong University of Science and Technology, Wuhan, China; ^2^ Center of Reproductive Medicine, Tengzhou Central People's Hospital, Tengzhou, China; ^3^ Center of Reproductive Medicine, The No.1 Hospital of Wuhan, Wuhan, China; ^4^ Wuhan Tongji Reproductive Medicine Hospital, Wuhan, China

**Keywords:** unexplained asthenozoospermia, primate specific, epididymal miRNA, sperm maturation, epigenetic basis

## Abstract

Asthenoszoopermia, characterized by reduced sperm motility, is one of the primary forms of male infertility. Whereas most cases were diagnosed into unexplained asthenozoospermia (UA) because the etiology cannot be identified. In animal models, epigenetic dysregulation in epididymis can impair sperm maturation and result in asthenozoospermia. However, researches of epididymal epigenetic regulation on humans are impeded by the difficulty in obtaining epididymal tissues. We previously identified cell-free seminal microRNAs predominately derived from epididymis in human ejaculate. In the present study, these microRNAs were used to screen and validate the microRNA dysregulation in men with UA, which were divided into screening set and validation set. The expression of five miRNAs (miR-891b, miR-892b, miR-892a, miR-888 and miR-890) was found and confirmed to be dysregulated in men with UA. Interestingly, these five miRNAs belong to a primate-specific miRNA cluster located on the X chromosome with epididymis specific expression. Moreover, obvious coherent dysregulation of these miRNAs were observed in 13% men with UA. Regression analysis demonstrated that levels of these miRNAs were significantly correlated with progressive sperm motility. Functions and pathways of predicted target genes of this cluster suggested its role in sperm maturation. Dysregulation of this miRNA cluster might be an epigenetic basis for some patients with UA. We also showed a noninvasive and feasible approach to get epigenetic information of human epididymis.

## INTRODUCTION

Infertility affects one in seven couples with about 50% of cases caused by male problems [1]. Asthenoszoopermia, characterized by reduced sperm progressive motility, is a common cause of male infertility [2, 3]. The present reference range for this condition is <32% progressively motile spermatozoa in fresh ejaculate [4]. The impaired sperm progressive motility causes infertility because forward motility is essential for the migration of sperm in the female reproductive tract, the penetration of cumulus oophorus, and the fertilization. Unfortunately, the etiology for many patients with asthenozoospermia is still unknown and is termed unexplained asthenozoospermia (UA). The only available treatment option for these patients is assisted reproductive technology, which bypasses the natural selection with the risk of transmitting genetic and epigenetic anomalies. Therefore, revealing the genetic and epigenetic causes for asthenozoospermia can not only provide the missing etiologic factors and appropriate counseling, but also a rational basis for the development of future etiology-based prevention and therapies.

The defective epididymal function should be the etiology of many patients with UA, because during natural sperm maturation, progressive motility is acquired during epididymal transit. Epididymis is a single highly coiled duct with extreme long length (3 to 6 m in humans). The differential expression of genes along the epididymis is finely regulated to maintain the appropriate epididymal microenvironment of different regions. Epigenetic control, in particular miRNAs posttranscriptional pathway, of gene expression may act as key regulators of sperm maturation in the epididymis [5, 6]. Upon inhibiting biogenesis of epididymal miRNAs by knockout Dicer1 of mouse epididymis, the sperm showed significantly reduced ability of progressive motility and fertilizing the oocyte [7]. Dozens of epididymal miRNA profiles are transmitted to mouse sperm which are extensively modified during epididymal maturation under physiological conditions [8]. Abnormal histone methylation induced alteration of epididymal genes expression may also result in significantly reduced motile sperm [9]. However, most efforts have been devoted to reveal the epigenetic control of sperm maturation these are based on animal models. Researches on epigenetic basis of human sperm maturation are impeded by the difficulties in obtaining epididymal tissues. Usually, epididymis puncture is used to obtain sperm for assisted reproductive technologies. A more accessible and reliable way to obtain epigenetic information of the human epididymis is thus desired.

Extracellular nucleic acids existing in seminal plasma, that is, cell-free seminal RNAs and DNA, may be a feasible noninvasive approach to obtain epigenetic information from human epididymis. Cell-free RNAs and DNA have been found in various human biofluids and widely used as biomarkers in liquid biopsy for disease diagnosis and research [10, 11]. These extracellular nucleic acids can be released from the dying cells or actively secreted by living cells, thus can provide gene expression and epigenetic information of the cells or tissues from which the biofluid is secreted. Previously, we have found the presence of cell-free seminal RNAs at high concentration in the human ejaculate [12]. Further researches demonstrated that gene expression and epigenetic information of testis can be obtained from cell-free seminal RNAs and DNA methylation [13–17]. Cell-free seminal RNA even was more accurate than single testis biopsy for the identification of azoospermia with Sertoli-cell-only [18].

As for the cell-free seminal miRNA (cfs-miRNA) and epididymis, abundant cfs-miRNAs were identified [19] and they kept fairly stable mainly by binding with protein complexes [20]. However, the human ejaculate is a mixture of secretions from male reproductive organs. Given that the secretion from epididymis only accounts for less than 5% of the volume of human semen, the noise for the quantification of miRNAs non-specific for epididymis from cfs-miRNA would be large. We thus identified cfs-miRNAs predominately derived from epididymis by combining the comparison between normozoospermic participants and vasectomized individuals, and miRNA expression database [21]. Totally 13 miRNAs showed much lower level (even undetectable) in vasectomized men than in normozoospermic individuals, and also showed specifically or preferentially expressed in epididymis, notably 9 of them were located on the X-chromosome which contains varies genes responsible for male reproduction and sperm maturation [22, 23]. Levels of these cfs-miRNAs could reflect their levels in epididymis, thus could be used as reliable and feasible way to obtain epigenetic information of human epididymis.

The present study screened and validated the dysregulated miRNAs in men with UA from the 13 cfs-miRNAs predominately derived from the epididymis. Then, associations between levels of 5 validated miRNAs and sperm motility was determined. Finally, bioinformatics analysis subjected to miRNA target genes were performed, aiming to probe epigenetic aetiology or mechanism for UA.

## RESULTS

### Characteristics of participants

Totally 69 men with UA were chosen from 403 patients with asthenozoospermia after system examination. These men with UA were divided into screening set (n=22) and validation set (n=47). Same amount of normozoospermic donors were chosen and included in each set. Apart from significantly lower percentage of progressively motile sperm in men with UA compared with normozoospermic donors, age, volume of seminal plasma and sperm concentration present no difference between two groups (Table 1).

**Table 1:** Characteristics of asthenozoospermic patients and normozoospermic donors

Study set	Group	Age (years)	Volume (ml)	Concentration(10^6^/ml)	Progressivemotility (%)
Screening	Asthenozoospermia	32.4±6.0	2.27±0.40	51.6±25.93	17.83±7.21*
	Normozoospermia	32.2±5.8	2.42±0.64	53.3±21.61	49.73±10.24
Validation	Asthenozoospermia	33.73±6.63	3.16±1.26	65.60±39.96	13.52±7.51*
	Normozoospermia	33.00±7.22	2.77±1.10	69.87±43.79	49.08±12.54

All variables were presented as mean ± SD, differences between asthenozoospermic patients and normozoospermic donors were analyzed using t-test.

*P*=0.000, compared with normozoospermic donors.

### Screening miRNA candidates

**Figure 1:**
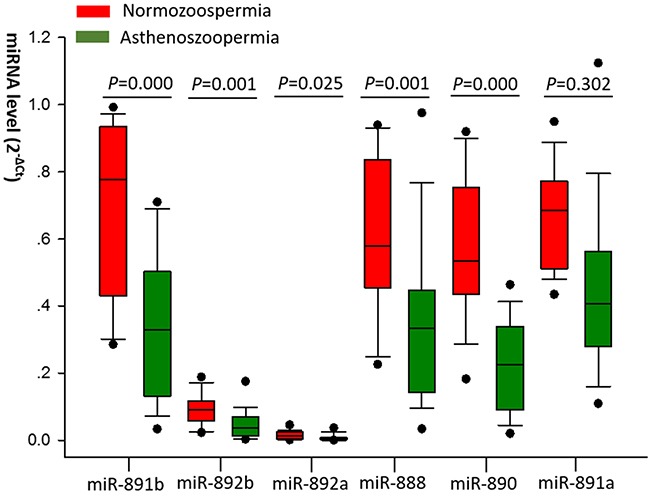
The different expression levels of miRNAs in asthenozoospermic patients and normozoospermic donors in screening set The green boxplot represent normozoospermia, red boxplot represent asthenozoospermia. Expression levels of the miRNAs (2^−ΔCt^ scale at Y-axis) were normalized by the mean Ct value of miR-30d and miR-93. Higher expression levels of these miRNAs have a higher 2^−ΔCt^ scale. The line within the box represents the median value, the line above and under the box represent the marginal value and the solid plots represent outliers. Mann-Whitney U test was performed to determine statistical significance.

From the 13 cfs-miRNAs previously identified to be predominately derived from the epididymis [21], five miRNAs (miR-891b, miR-892b, miR-892a, miR-888 and miR-890), were significantly down-regulated (P=0.000, 0.001, 0.025, 0.001, 0.000, respectively) in men with UA, as compared with the normozoospermic control donors (Figure 1). Whereas no difference of other miRNA levels was observed in patients with UA and normozoospermic donors.

Interestingly, these five dysregulated miRNAs belong to an X-linked epididymis-specific miRNA cluster (miR-888 cluster) [5, 24]. However, a member of this cluster, miR-891a, which locates relatively far from other five members [20], was not significantly dysregulated in the UA group (P=0.302). Genomic location of miR-888 cluster members was shown in Supplementary Figure 1.

### Validating the dysregulated miRNAs

**Figure 2:**
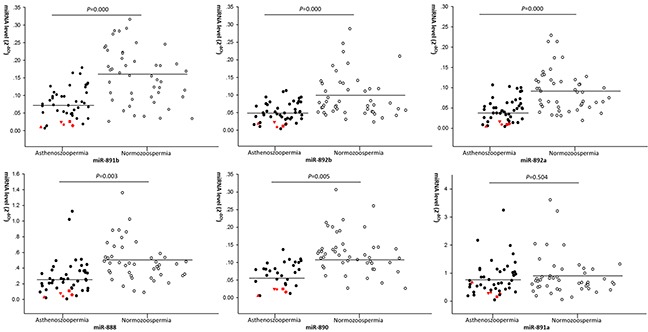
The different expression levels of miRNAs in asthenozoospermic patients and normozoospermic donors in larger scale validation set The solid plot represent asthenozoospermia, hollow plot represent normozoospermia. Expression levels of the miRNAs (2^−ΔCt^ scale at Y-axis) were normalized by the mean Ct value of miR-30d and miR-93. Higher expression levels of these miRNAs have a higher 2^−ΔCt^ scale. The line represents the median value. Mann-Whitney U test was performed to determine statistical significance. The five asthenozoospermic patients (▲ ▼ ■ ♦ ★) with obvious dysregulation of five validated miRNAs (fold-change>2) were presented.

All the six members of the miR-888 cluster were then quantified and compared in the validation set with relatively larger sample size (n=47 for both UA patients and healthy donors). Results similar to the screen step was observed. Levels of miR-891b, miR-892b, miR-892a, miR-888 and miR-890 were significantly lower in UA patients compared with normozoospermic donors (*P*=0.000, 0.000, 0.000, 0.003, 0.005, respectively), and no significant difference was observed of miR-891a level between two groups (*P*=0.504) (Figure 2).

### Patients with obvious coherent dysregulations of 5 validated miRNAs

After identifying 5 members of miR-888 cluster (miR-891b, miR-892b, miR-892a, miR-888 and miR-890) dysregulated in UA patients, we analyzed characteristics of these 5 miRNAs dysregulation in validation set. Interestingly, obvious coherent dysregulations (fold-change > 2) of these miRNAs were observed in at least 5 patients with UA (Table 2, Figure 2). We then went back to check results of the screening step, and 4 patients with obvious coherent dysregulations of the 5 miRNAs were also found (Table 2, Supplementary Figure 2). In summary, about 13% (9/69) UA patients showed obvious coherent dysregulations of these 5 validated miRNAs.

**Table 2:** Characteristics of patients with obvious coherent dysregulations of 5 validated miRNAs

Study set	Subject symbol	Age (years)	Volume (ml)	Concentration(10^6^/ml)	Progressivemotility (%)
Screening	# & **+ ×**	33.5±3.6	2.18±0.20	47.2±18.4	15.69±8.42
	#	39	2.0	35.00	17.55
	**&**	29	2.0	46.51	25.97
	**+**	34	2.2	77.32	2.52
	**×**	32	2.5	30.15	16.71
Validation	▲ ▼ ■ ♦ ★	31.80±2.95	3.26±1.69	45.04±33.95	10.94±5.49
	▲	31	2.6	103.66	2.05
	▼	33	6.1	29.49	10.29
	■	34	3	37.85	14.66
	♦	34	1.6	16.25	16.13
	★	27	3	37.95	11.56

All variables were presented as mean ± SD

### Relationship between levels of dysregulated cfs-miRNAs and sperm motility

**Figure 3:**
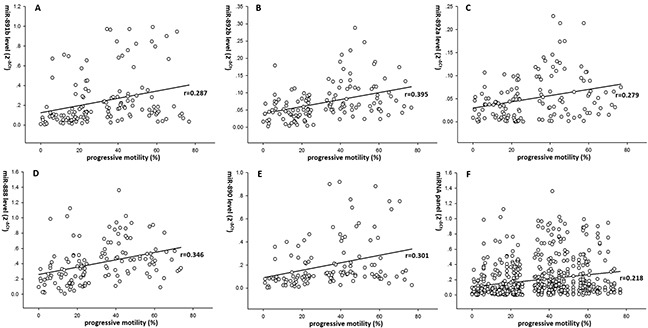
Correlation between the validated epididymal miRNAs and spermatozoa motility Expression levels of these miRNAs (2^−ΔCt^ scale at Y-axis) were normalized by the mean Ct value of miR-30d and miR-93. The results suggested that miR-891b, miR-892b, miR-892a, miR-888 and miR-890 were correlated with sperm progressive motility separately (r=0.287, 0.395, 0.279, 0.346 and 0.301, p=0.001, 0.000, 0.001, 0.000 and 0.001 respectively) **(A-E).** Levels of miR-891b/miR-892b/miR-892a/ miR-888/miR-890 combination (miRNA panel) was also significantly correlated with sperm progressive motility (r=0.218, p=0.000) **(F).**

**Figure 4:**
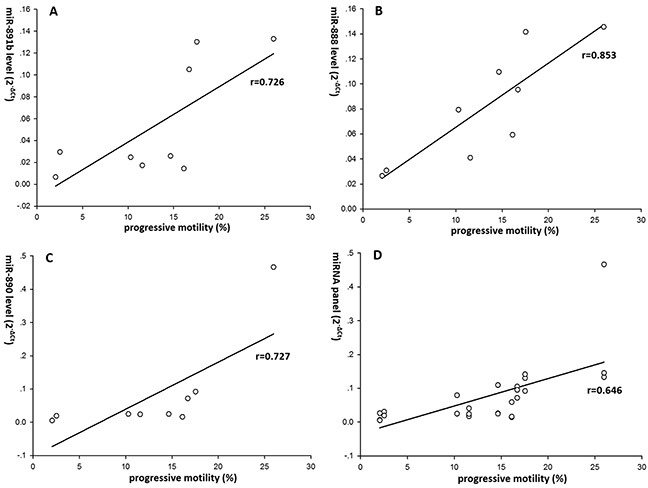
Correlation between the validated epididymal miRNAs and spermatozoa motility in 9 patients with obvious coherent dysregulations of the 5 validated miRNAs Expression levels of these miRNAs (2^−ΔCt^ scale at Y-axis) were normalized by the mean Ct value of miR-30d and miR-93. The results suggested that levels of miR-891b **(A)**, miR-888 **(B)**, miR-890 **(C)** and miRNA panel **(D)** including miR-891b/miR-888/miR-890 in 9 patients with obvious coherent dysregulations of 5 validated miRNAs showed stronger correlation with sperm progressive motility (r=0.726, 0.853, 0.727 and 0.646, p=0.027, 0.003, 0.026 and 0.000, respectively).

Regression analyzes were performed to investigate the association between the expression levels of miR-891b, miR-892b, miR-892a, miR-888, miR-890 and sperm motility. We found that miRNA level of miR-891b, miR-892b, miR-892a, miR-888 and miR-890 were correlated with sperm progressive motility separately (r=0.287, 0.395, 0.279, 0.346 and 0.301, p=0.001, 0.000, 0.001, 0.000 and 0.001 respectively) (Figure 3A-3E). Levels of miR-891b/miR-892b/miR-892a/miR-888/miR-890 combination (miRNA panel) was also significantly correlated with sperm progressive motility (r=0.218, p=0.000) (Figure 3F). Moreover, levels of miR-891b, miR-888, miR-890 and miRNA panel including miR-891b/miR-888/miR-890 in 9 Patients with obvious coherent dysregulations of 5 validated miRNAs showed stronger correlation with sperm progressive motility (r=0.726, 0.853, 0.727 and 0.646, p=0.027, 0.003, 0.026 and 0.000, respectively) (Figure 4).

### Gene ontology and pathway analysis based on miRNA-targeted genes

**Figure 5:**
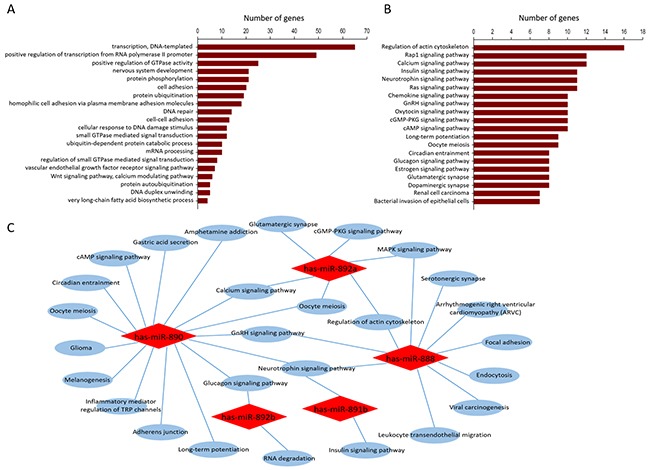
Gene ontology and pathway analysis based on validated miRNA-targeted genes Top 20 of GOs and KEGG pathways enrichment predicted by the dysregulated miRNAs are presented **(A, B)**, possible functional network of selected miRNAs are constructed **(C).**

A total of 460 target genes were identified for the 5 validated epididymal miRNAs. Functions and pathways of predicted target genes of these miRNAs were gained and filtered according to p-value (*P* < 0.05 were included) and the top 20 GO terms based on biological process and pathway enrichment were presented (Figure 5A). GO analysis suggested altered biological process related to positive regulation of DNA-templated transcription, GTPase activity and cell adhesion. In order to find out potential biological pathways controlled by these involved miRNAs, KEGG pathway analysis were performed for predicted target genes. These target genes were also enriched in various biological pathways, such as regulation of actin cytoskeleton, Rap1 signaling pathway and calcium signaling pathway (Figure 5B). Furthermore, functional network of biological pathway subjected to predicted target genes showed several intersecting pathways of selected miRNAs, such as regulation of actin cytoskeleton, calcium signaling pathway, neurotrophin signaling pathway and MAPK signaling pathway (Figure 5C).

## DISCUSSION

The present study investigated 13 cfs-miRNAs predominantly derived from epididymis based on our previous work [21] in seminal plasma of UA patients, and firstly identified the dysregulation of an X-linked epididymis specific miRNA cluster in UA patients when compared with the normozoospermic donors. Levels of these miRNAs were significantly correlated with sperm motility. Interestingly, obvious coherent dysregulations of five members of this cluster were observed in about 13% patients with UA, levels of miR-891b, miR-888 and miR-890 in these 9 patients with obvious coherent dysregulations of the 5 miRNAs showed stronger correlation with sperm motility. GO and pathway analysis also indicated this cluster may involve in sperm maturation. Given the pivotal role of epididymis in sperm maturation, this epididymal miRNA cluster might be an epigenetic mediator of sperm epididymal maturation, dysregulation of these miRNAs might be an epigenetic basis in some patients with UA. On the other hand, the present study provided a noninvasive approach to get epigenetic information of epididymis, of which the tissue usually cannot be obtained in patients with male infertility.

The significantly lower levels of miR-888 cluster in seminal plasma of UA patients indicate their dysregulation in the epididymis. cfs-miRNAs could reflex the miRNA expression profile of bilateral or whole organs from which the semen plasma secreted, if no obstruction exist. Although the secretion from epididymis only accounts <5% of the seminal plasma, all 13 cfs-miRNAs investigated in the present study have been certified to be predominantly derived from the epididymis [21]. These miRNAs were undetectable or showed significant lower levels in seminal plasma of vasectomized men when compared with normozoospermic individuals. And all these miRNAs were epididymis specific or preferentially expressed. Therefore, levels of these miRNAs in seminal plasma could reflex their expression in epididymis, and be used for a feasible and noninvasive approach to get epigenetic information of human epididymis. One may argue that the variation of the volume of epididymal secretion may exist in different individuals, which may lead to the different levels of miRNAs in seminal plasma. Individual variation of the volume of epididymal secretion may exist, although no convinced research is available. However, in the present study, from the 13 miRNAs which are all predominantly derived from epididymis, only 5 miRNAs belonging to a cluster showed significantly lower levels in UA patients when compared with normozoospermic individuals, whereas other 8 miRNAs showed similar levels. Previous study also showed similar levels of parameters indicating the secretory capacity of the epididymis, including neutral α-glucosidase, free carnitine, total carnitine and acetylated carnitine, between asthenozoospermic patients and normozoospermic individuals [25].

The obvious coherent dysregulation of five members of the miR-888 cluster in 13% men with UA and function analysis of their targeted genes suggested that dysregulation of these miRNAs might be an epigenetic basis for some patients with UA. Unlike the experiment on animal models, in which coherent results can be observed in all or most animals in the same group, differential results can be usually observed in person with the same disease due to individual difference. In the present study, although levels of the five members of the miR-888 cluster in the group with UA were significantly lower than the normozoospermic group, obvious individual difference was observed in both groups. However, obvious coherent dysregulation of the five miRNAs was observed in 9 out of 69 men with UA. Dysregulation of this miRNA cluster may be an epigenetic etiology for these patients. This epididymis specific miRNA cluster may regulate target gene expression within somatic epithelial cells in a region-specific manner. Upon releasing into epididymal luminal, they may interact with maturing sperm and epididymal epithelial cells in a well-orchestrated manner [26]. To better understand the underlying molecular functions of miR-888 cluster, GO and KEGG pathway analysis subjected to their target genes were performed. Generally, these target genes are enriched in diverse biological process ranging from fundamental cellular operation to protein modification and cell-cell communication. Notably, GTPase and MAPKs are key regulators in cell signaling, involved in diverse cellular process such as growth, differentiation, stress response and apoptosis, they are suggested to have an important role in maintaining sperm motility [27, 28]. In addition, calcium can regulate sperm motility initiation and dysregulation of calcium pathway might be a molecular mechanism of asthenozoospermia [29]. Finally, actin cytoskeleton is key regulator of multiple cellular processes such as maintenance of cell shape, membrane trafficking and cell locomotion [30]. Dysregulation of actin cytoskeleton might be involved in idiopathic asthenozoospermia [31]. In summary, our results suggested multiple physiological roles of miR-888 cluster on human epididymis, especially on epididymal sperm maturation. Indeed, the regression analysis demonstrated that levels of these miRNAs were significantly correlated with progressive sperm motility, which is acquired during sperm transit in the epididymis.

Apart from regulation of the target gene expression, miRNAs themselves are subjected to sophisticated regulation either by internal inherent factors or external environmental factors. In the former case, a major control of tissue-specific or development-specific expression of miRNAs is conducted via regulation of miRNA gene transcription [32], such as common genetic variants (mutation of SNP, alterations in DNA methylation and copy number, etc.) in miRNAs genes [33, 34]. Meanwhile, miRNA expression can be regulated by external environmental factors, such as heat stress, environmental pollutants exposure, infections and immunological factors, etc. [35]. Given the fact that all UA men recruited in this study were restricted to least disturbance of environmental factors, inherent problem might be the primary cause responsible for dysregulation of miR-888 cluster in UA men. In spite of great efforts we devoted in investigating inherent regulation of the primate-specific miRNA cluster expression, further studies are hindered by difficulties in obtaining human epididymal tissues or epithelial cells, more studies are needed to shed new light on inherent basis of dysregulation of miR-888 cluster in UA men.

In summary, we firstly identified the dysregulation of an X-linked epididymis specific miRNA cluster in UA patients when compared with the normozoospermic donors. Obvious coherent dysregulations of these miRNAs were present in about 13% UA patients, levels of miR-891b, miR-888 and miR-890 in these 9 patient with obvious coherent dysregulations of the 5 miRNAs showed stronger correlation with sperm motility. It may be epigenetic mediator of sperm epididymal maturation. Dysregulation of this epididymis specific miRNA cluster might be an epigenetic basis for some patients with UA, thus providing a potential biomarker for the etiology diagnosis of asthenozoospermia. Given that the effect of a therapy on sperm motility can usually be observed after one month or more, the recovery of the miR-888 cluster expression would occur at early stage of an effective therapy and be helpful in the prognosis. Moreover, our work provided a noninvasive and feasible approach to get epigenetic information of epididymis, bypassing the obstacle in obtaining epididymal tissues of infertile men, thus provided a broad foundation for further investigation on molecular basis of male infertility.

## MATERIALS AND METHODS

### Study population

Asthenozoospermic patients were recruited from Wuhan Tongji Reproductive Medicine Hospital. Men with UA were included by the following criteria: There was no evidence of anatomical abnormalities of the genital tract, including varicocele, nor was there a history of cryptorchidism, mumps, eating raw cottonseed oil, testicular torsion, hyperpyrexia, genital tract infection, drug addiction, occupational exposure of chemical material and hyperthermia environment, etc. The culture of semen was negative, including Escherichia coli, Staphylococcus aureus, Streptococcus, Neisseria gonorrhoeae, Ureaplasma urealyticum, Chlamydia, Mycoplasma detection. The hormonal serum profile (gonadotropins, prolactin, testosterone, estradiol, follicle stimulating hormone and luteinizing hormone) was normal. Patients with abnormal semen PH or liquefaction were also excluded. The absence of known genetic causes (chromosome abnormality, and Y microdeletion), immune disease (including anti-sperm antibody), and other systemic diseases was also verified. WHO guidelines for human semen parameters and reference values were used for asthenozoospermia and normozoospermia [4]. Healthy donors (normozoospermia) were recruited as described previously [12].

This study was approved by our institutional review board, and written informed consent was obtained from all individuals. Clinical investigation was conducted according to the principles expressed in the Declaration of Helsinki.

### Study design

Two steps, screening miRNA candidates and validation, were included. In the screening step, the 13 previously identified cfs-miRNAs (miR-892a, miR-888, miR-891b, miR-890, miR-892b, miR-891a, miR-421, miR-200b, miR-181c, miR-221, miR-660, miR-187 and miR-200c) [21] were quantified and compared in cohort of men with UA and matched healthy donors (n=22 for each group). Dysregulated miRNAs were further validated in another cohort of UA patients and matched healthy donors (n=47 for each group).

### Sample collection and procession

Semen samples were obtained by masturbation after 3-5 days of sexual abstinence and were allowed to liquefy within 30 min at 37°C. Samples were centrifuged twice at 4°C (1,600 g for 10 min, then 16,000 g for 10 min) to harvest cell-free seminal plasma as previously described [12]. The supernatant (seminal plasma) was carefully collected and immediately frozen in liquid nitrogen for RNA isolation.

### RNA isolation

Total RNA was extracted as described previously with minor modification [12]. In brief, untreated seminal plasma after thawing was immediately confused with Trizol LS (Invitrogen, Carlsbad, USA) at a higher concentration (0.2ml untreated seminal plasma with 0.8ml Trizol LS) to ensure enough quantity for consequent miRNA cDNA synthesis. All procedures were followed according to the manufacturer's instruction.

The purity of total RNA was checked using an ultraviolet spectrophotometer (Biometra, Göttingen, Germany) at 260 nm and 280 nm, and the RNA concentration was calculated using the OD260.

### Quantitative real-time PCR (qRT-PCR)

Before qRT-PCR, DNase I (Takara, Japan) treatment was carried out according to the manufacturer's instructions to remove any contaminating DNA. miRNAs were then converted into cDNA using the One Step PrimeScript^®^ miRNA cDNA Synthesis Kit (Code: D350A, Takara, Japan), and then subjected to real-time PCR amplification using SYBR Premix Ex Taq^TM^ II (Perfect Real Time) (Code: RR820A, Takara, Japan) according to the manufacturer's instruction. A 20 μl reaction mixture containing 10 μl of SYBR Premix Ex Taq II (Tli RNaseH Plus) (2×), 0.8 μl of each primer (0.4 μM), 2 μl of template DNA and 6.4 μl of ddH_2_O were placed in the real-time PCR apparatus. Reaction mixtures were thermally cycled under the following conditions: Initial denaturation at 95°C for 30 sec; Followed by 40 cycles at 95°C for 5 sec, 60°C for 20 sec, and then a melting curve was generated when thermally cycled once at 95°C for 30 sec, 60°C for 30 sec and 95°C for 30 sec. All real-time PCR was performed in LightCycler (Roche, Switzerland). The quantification cycle (Ct) was automatically calculated with instrument default threshold settings (adaptive baseline). Suggested endogenous miRNA controls (miR-30d, miR-93) were selected to normalizing miRNA profiles of seminal plasma [36, 37]. Primers for all miRNAs are given in Supplementary Table 1. Mean Cq of the endogenous miRNA controls was applied to calculate the ΔCt of target miRNA (formula: mean Ct _target miRNA_ -mean Ct _controls_).

### Bioinformatics analysis

TargetScan (http://www.targetscan.org/) and miRa-nda (http://www.microrna.org/) were used to predict the intersection of target genes of miRNAs. Enrichment of predicted target genes was performed using Gene Ontology (GO) (http://www.geneontology.org/) and Kyoto Encyclopedia of Genes and Genomes (KEGG) (http://www.genome.jp/kegg/). GO terms and KEGG Pathway annotation of the miRNA target genes was conducted with Database for Annotation, Visualization, and Integrated Discovery (DAVID) (http://david.abcc.ncifcrf.gov/), Fisher's exact test was used to select significant GO terms and KEGG pathway, the false discovery rate (FDR) was used to correct the p value. GO terms and pathways with p value of < 0.05 and FDR value of < 0.05 were chosen. Cytoscape 3.3.0 was used to construct the possible functional network of selected miRNAs.

### Statistical analysis

Statistical analyses were performed using SPSS software 19.0 package (IBM corp., New York, USA). Continuous variables were presented as the means ± SD and analysis of variance (ANOVA) or t-test was used to calculate the difference between groups. Expression levels of miRNAs were calculated using the 2^−ΔCt^ method and compared using the Mann-Whitney U test. The correlations between the expression levels and percentages of progressively motile sperm were assessed with the Pearson's correlation coefficient. P<0.05 was considered statistically significant.

## SUPPLEMENTARY MATERIALS



## Abbreviations

UA: unexplained asthenozoospermia
miRNA: microRNA
cfs-miRNA: cell-free seminal miRNA
qRT-PCR: quantitative real-time PCR

## References

[1] Mascarenhas MN, Flaxman SR, Boerma T, Vanderpoel S, Stevens GA (2012). National, regional, and global trends in infertility prevalence since 1990: a systematic analysis of 277 health surveys. PLoS Med.

[2] Thonneau P, Marchand S, Tallec A, Ferial ML, Ducot B, Lansac J, Lopes P, Tabaste JM, Spira A (1991). Incidence and main causes of infertility in a resident population (1,850,000) of three French regions (1988-1989). Hum Reprod.

[3] Curi SM, Ariagno JI, Chenlo PH, Mendeluk GR, Pugliese MN, Sardi Segovia LM, Repetto HE, Blanco AM (2003). Asthenozoospermia: analysis of a large population. Arch Androl.

[4] WHO (World Health Organization) (2010). WHO laboratory manual for the examination and processing of human semen, 5th Ed.

[5] Belleannee C, Calvo E, Thimon V, Cyr DG, Legare C, Garneau L, Sullivan R (2012). Role of microRNAs in controlling gene expression in different segments of the human epididymis. PLoS One.

[6] Belleannee C, Legare C, Calvo E, Thimon V, Sullivan R (2013). microRNA signature is altered in both human epididymis and seminal microvesicles following vasectomy. Hum Reprod.

[7] Bjorkgren I, Gylling H, Turunen H, Huhtaniemi I, Strauss L, Poutanen M, Sipila P (2015). Imbalanced lipid homeostasis in the conditional Dicer1 knockout mouse epididymis causes instability of the sperm membrane. FASEB J.

[8] Nixon B, Stanger SJ, Mihalas BP, Reilly JN, Anderson AL, Tyagi S, Holt JE, McLaughlin EA (2015). The microRNA signature of mouse spermatozoa is substantially modified during epididymal maturation. Biol Reprod.

[9] Brinkmeier ML, Geister KA, Jones M, Waqas M, Maillard I, Camper SA (2015). The histone methyltransferase gene absent, small, or homeotic discs-1 like is required for normal hox gene expression and fertility in mice. Biol Reprod.

[10] Hui L, Bianchi DW (2011). Cell-free fetal nucleic acids in amniotic fluid. Hum Reprod Update.

[11] Schwarzenbach H, Hoon DS, Pantel K (2011). Cell-free nucleic acids as biomarkers in cancer patients. Nat Rev Cancer.

[12] Huang S, Li H, Ding X, Xiong C (2009). Presence and characterization of cell-free seminal RNA in healthy individuals: implications for noninvasive disease diagnosis and gene expression studies of the male reproductive system. Clin Chem.

[13] Li H, Wu C, Gu X, Xiong C (2012). A novel application of cell-free seminal mRNA: non-invasive identification of the presence of germ cells or complete obstruction in men with azoospermia. Hum Reprod.

[14] Pansa A, Sirchia SM, Melis S, Giacchetta D, Castiglioni M, Colapietro P, Fiori S, Falcone R, Paganini L, Bonaparte E, Colpi G, Miozzo M, Tabano S (2014). ESX1 mRNA expression in seminal fluid is an indicator of residual spermatogenesis in non-obstructive azoospermic men. Hum Reprod.

[15] Wu C, Ding X, Li H, Zhu C, Xiong C (2013). Genome-wide promoter methylation profile of human testis and epididymis: identified from cell-free seminal DNA. BMC Genomics.

[16] Wu C, Ding X, Tan H, Li H, Xiong C (2016). Alterations of testis-specific promoter methylation in cell-free seminal deoxyribonucleic acid of idiopathic nonobstructive azoospermic men with different testicular phenotypes. Fertil Steril.

[17] Li HG, Huang SY, Zhou H, Liao AH, Xiong CL (2009). Quick recovery and characterization of cell-free DNA in seminal plasma of normozoospermia and azoospermia: implications for non-invasive genetic utilities. Asian J Androl.

[18] Turek PJ, Johnson MH (2016). A seminal molecular marker for sperm presence in non-obstructive azoospermia?. Reprod Biomed Online.

[19] Weber JA, Baxter DH, Zhang S, Huang DY, Huang KH, Lee MJ, Galas DJ, Wang K (2010). The microRNA spectrum in 12 body fluids. Clin Chem.

[20] Li H, Huang S, Guo C, Guan H, Xiong C (2012). Cell-free seminal mRNA and microRNA exist in different forms. PLoS One.

[21] Hu L, Wu C, Guo C, Li H, Xiong C (2014). Identification of microRNAs predominately derived from testis and epididymis in human seminal plasma. Clin Biochem.

[22] Sutton KA, Wilkinson MF (1997). The rapidly evolving Pem homeobox gene and Agtr2, Ant2, and Lamp2 are closely linked in the proximal region of the mouse X chromosome. Genomics.

[23] Stouffs K, Lissens W (2012). X chromosomal mutations and spermatogenic failure. Biochim Biophys Acta.

[24] Li J, Liu Y, Dong D, Zhang Z (2010). Evolution of an X-linked primate-specific micro RNA cluster. Mol Biol Evol.

[25] Zopfgen A, Priem F, Sudhoff F, Jung K, Lenk S, Loening SA, Sinha P (2000). Relationship between semen quality and the seminal plasma components carnitine, alpha-glucosidase, fructose, citrate and granulocyte elastase in infertile men compared with a normal population. Hum Reprod.

[26] Belleannee C (2015). Extracellular microRNAs from the epididymis as potential mediators of cell-to-cell communication. Asian J Androl.

[27] Almog T, Lazar S, Reiss N, Etkovitz N, Milch E, Rahamim N, Dobkin-Bekman M, Rotem R, Kalina M, Ramon J, Raziel A, Breitbart H, Seger R (2008). Identification of extracellular signal-regulated kinase 1/2 and p38 MAPK as regulators of human sperm motility and acrosome reaction and as predictors of poor spermatozoan quality. J Biol Chem.

[28] Marchiani S, Tamburrino L, Ricci B, Nosi D, Cambi M, Piomboni P, Belmonte G, Forti G, Muratori M, Baldi E (2014). SUMO1 in human sperm: new targets, role in motility and morphology and relationship with DNA damage. Reproduction.

[29] Pereira R, Sa R, Barros A, Sousa M (2017). Major regulatory mechanisms involved in sperm motility. Asian J Androl.

[30] Pollard TD, Cooper JA (2009). Actin, a central player in cell shape and movement. Science.

[31] Salvolini E, Buldreghini E, Lucarini G, Vignini A, Lenzi A, Di Primio R, Balercia G (2013). Involvement of sperm plasma membrane and cytoskeletal proteins in human male infertility. Fertil Steril.

[32] Krol J, Loedige I, Filipowicz W (2010). The widespread regulation of microRNA biogenesis, function and decay. Nat Rev Genet.

[33] Zhao H, Xu J, Zhao D, Geng M, Ge H, Fu L, Zhu Z (2016). Somatic mutation of the SNP rs11614913 and its association with increased MIR 196A2 expression in breast cancer. DNA Cell Biol.

[34] Aure MR, Leivonen SK, Fleischer T, Zhu Q, Overgaard J, Alsner J, Tramm T, Louhimo R, Alnaes GI, Perala M, Busato F, Touleimat N, Tost J (2013). Individual and combined effects of DNA methylation and copy number alterations on miRNA expression in breast tumors. Genome Biol.

[35] Chawla JP, Iyer N, Soodan KS, Sharma A, Khurana SK, Priyadarshni P (2015). Role of miRNA in cancer diagnosis, prognosis, therapy and regulation of its expression by Epstein-Barr virus and human papillomaviruses: with special reference to oral cancer. Oral Oncol.

[36] Meyer SU, Pfaffl MW, Ulbrich SE (2010). Normalization strategies for microRNA profiling experiments: a ‘normal’ way to a hidden layer of complexity?. Biotechnol Lett.

[37] Bargaje R, Hariharan M, Scaria V, Pillai B (2010). Consensus miRNA expression profiles derived from interplatform normalization of microarray data. RNA.

