# Supplementary material for: Dysregulation of an X-linked primate-specific epididymal microRNA cluster in unexplained asthenozoospermia
Source: Oncotarget. 2017 May 23;8(34):56839–49. doi: 10.18632/oncotarget.18076 (PMC5593606; doi:10.18632/oncotarget.18076)
Supplement: Supplementary file 1 [file oncotarget-08-56839-s001.pdf]

Dysregulation of an X-linked primate-specific epididymal microRNA cluster in unexplained asthenozoospermia

SUPPLEMENTARY MATERIALS

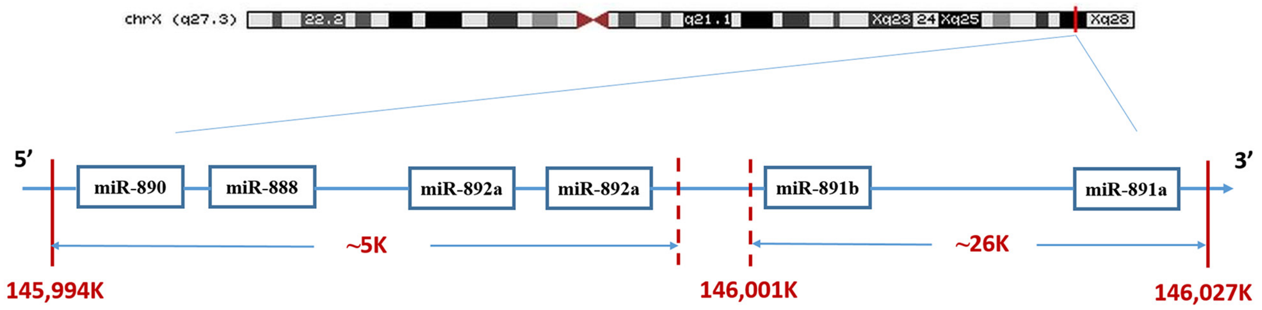

Supplementary Figure 1: Genomic location of miR-888 cluster.

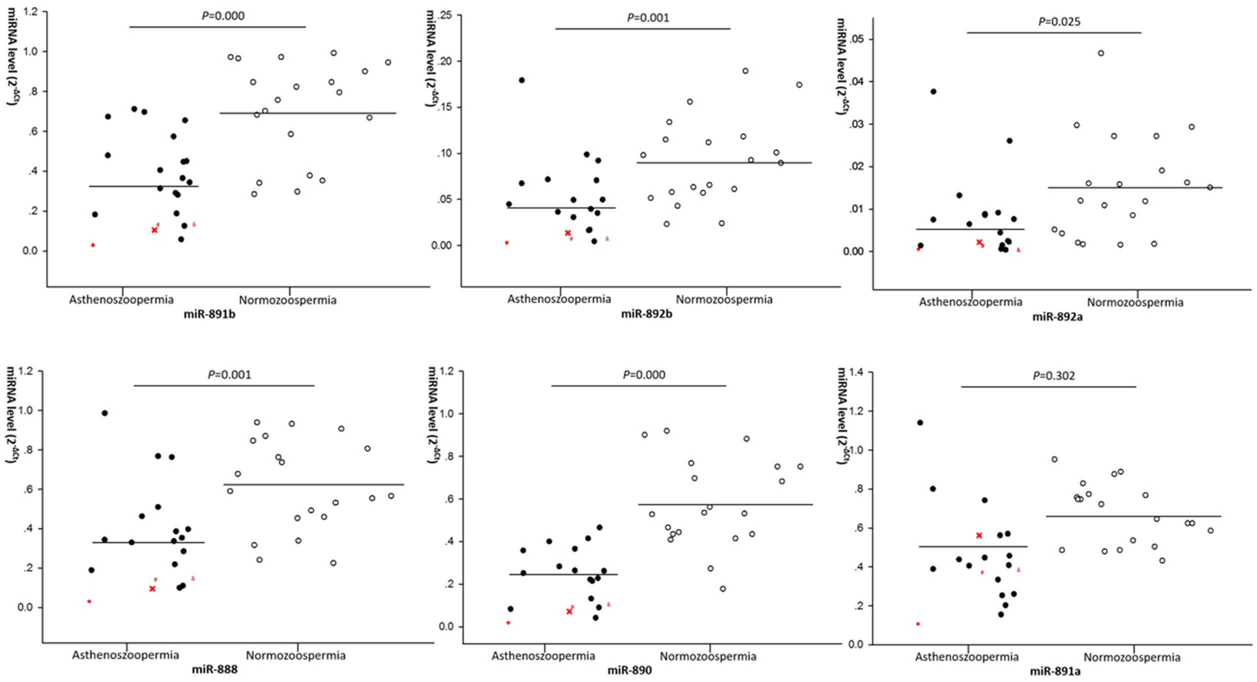

Supplementary Figure 2: The different expression levels of miRNAs in asthenozoospermic patients and normozoospermic donors in screening set. The solid plot represent asthenozoospermia, hollow plot represent normozoospermia. Expression levels of the miRNAs ($2^{-\Delta Ct}$ scale at Y-axis) were normalized by the mean Ct value of miR-30d and miR-93. Higher expression levels of these miRNAs have a higher $2^{-\Delta Ct}$ scale. The line represents the median value. Mann-Whitney U test was performed to determine statistical significance. The four asthenozoospermic patients (# & + ×) with obvious dysregulation of five validated miRNAs (fold-change>2) were presented.

Supplementary Table 1: Primers of epididymal miRNAs

miRNA	Primer
miR-30d	TGTAAACAUCCCCGACUGGAAG
miR-93	AGTGCTGTTCGTGCAGGTAG
miR-888	TACTCAAAAAGCTGTCAGTCA
miR-890	TACTTGGAAAGGCATCAGTTG
miR-891a	GCAACGAACCTGAGCCACTG
miR-891b	GCAACTTACCTGAGTCATTG
miR-892a	ACTGTGTCCTTTCTGCGTAG
miR-892b	CTGGCTCCTTTCTGGGTAGA
miR-421	CAACAGACATTAATTGGGCGC
miR-200b	TAATACTGCCTGGTAATGATGA
miR-181c	CATTCAACCTGTCGGTGAGT
miR-221	CTACATTGTCTGCTGGGTTTC
miR-660	CCCATTGCATATCGGAGTTG
miR-187	GTGTCTTGTGTTGCAGCCGG
miR-200c	ATACTGCCGGGTAATGATGGA
